# Low prevalence of depressive symptoms among stable patients on antiretroviral therapy in Johannesburg, South Africa

**DOI:** 10.1371/journal.pone.0203797

**Published:** 2018-09-25

**Authors:** Kate Shearer, Denise Evans, Barbara Xhosa, Kamban Hirasen, Craig Bracken, Kay Mahomed, Lawrence Long, Matthew P. Fox

**Affiliations:** 1 Health Economics and Epidemiology Research Office, Department of Internal Medicine, School of Clinical Medicine, Faculty of Health Sciences, University of the Witwatersrand, Johannesburg, South Africa; 2 Department of Psychiatry, School of Clinical Medicine, Faculty of Health Sciences, University of the Witwatersrand, Johannesburg, South Africa; 3 Right to Care, Johannesburg, South Africa; 4 Department of Epidemiology, Boston University School of Public Health, Boston, MA, United States of America; 5 Department of Global Health, Boston University School of Public Health, Boston, MA, United States of America; Imperial College London, UNITED KINGDOM

## Abstract

**Background:**

Depression is a leading cause of disability and may be associated with decreased adherence to ART. We sought to describe the prevalence of depressive symptoms and outcomes one year after screening among patients receiving ART at a large HIV Clinic in Johannesburg, South Africa.

**Methods:**

Adult (≥18) patients who had been on first-line ART between 6–18 months who could communicate in English were eligible. Depressive symptoms were evaluated using the Patient Health Questionnaire (PHQ)-9 and a score ≥10 indicated depression.

**Results:**

97 patients enrolled. Patients had been on ART for a median (IQR) of 8 (7–10) months, 61% were female, the median (IQR) age at enrollment was 38 (33–42) years, and the median (IQR) CD4 count at ART initiation was 154.5 (65–263) cells/mm^3^. 7 (7%) patients were found to have symptoms of depression; 4 (4%) had symptoms of moderate depression (PHQ score of 10–14) and 3 (3%) had symptoms of moderate/severe depression (PHQ score of 15–19). Women (10%) were more likely to have symptoms of depression than men (3%; prevalence difference [PD]: 7.5%; 95% confidence interval [CI]:-1.7%-16.8%); as were patients under the age of 30 (14%) compared to those 30–39 (4%; PD: -10.2; 95% CI: -29.4–9.0%) or ≥40 (9%; PD: -5.5%; -26.1%-15.2%), those with lower CD4 counts at ART initiation (<200 cells/mm^3^ vs ≥200 cells/mm^3^: 8% vs 3%; PD: 4.8%; 95% CI: -4.5%-14.0%), and those with high viral loads (>1000 copies/mL vs. <400 copies/mL: 40% vs. 5%; PD: 34.6%; 95% CI: -8.6%-77.6%). No relationship between depressive symptoms and retention in HIV care one year after screening was observed.

**Conclusions:**

We found a lower prevalence of depressive symptoms compared to findings from other HIV-positive populations in South Africa but more than one-third of patients with an elevated viral load had evidence of depression. Further research on the relationship between depression, adherence, and viral failure is warranted as this may present an opportunity for early interventions to improve treatment outcomes and reduce the need for second-line treatment.

## Introduction

Depression, defined by the World Health Organization (WHO) as “a common mental disorder, characterized by sadness, loss of interest or pleasure, feelings of guilt or low self-worth, disturbed sleep or appetite, feelings of tiredness, and poor concentration,” is a leading cause of morbidity globally.[1] The most recent Global Burden of Disease Study estimates that depressive disorders affect more than 250 million people worldwide, with over 150 million afflicted with major depressive disorder.[2] Currently, depression is the 5^th^ leading cause of Years Lived with Disability (YLDs) both globally and in South Africa.[2,3]

The 12-month prevalence of depression in the general population of South Africa is estimated to be 4.9% and lifetime prevalence is estimated at 9.7%.[4] However, people living with HIV tend to be disproportionately affected by mental health disorders, including depression, and many studies have documented much higher prevalence estimates among this population.[5] In the Western Cape, estimates of depression in people living with HIV have ranged from 14% to 62%.[6–8] A similar variation in estimates has been reported from other study sites across South Africa and at different points in the HIV care cascade. Among individuals testing positive for HIV, the prevalence of depressive symptoms has been estimated as 30% to 55% in Gauteng and KwaZulu-Natal, respectively.[9,10] Among people living with HIV, most of whom were not yet on antiretroviral therapy (ART), a study conducted across 5 provinces estimated the prevalence of depression at 40% and among patients recently initiated on ART in the Free State, the prevalence of depressive symptoms was 25%.[11,12]

Among people living with HIV who are on ART, research has shown that depression may be associated with decreased adherence[8,13,14] which in turn may result in poor immune response, increased risk of co-infections, the need for more expensive second- and third-line regimens, and potentially death. In this study, we aimed to estimate the prevalence of depressive symptoms among people living with HIV who had been on ART for at least 6 months. In addition, we describe one-year outcomes, including virologic response as a proxy marker for adherence and retention in care, among those individuals with and without depressive symptoms.

## Methods

### Study site

This study was conducted at the Themba Lethu HIV Clinic in Johannesburg, South Africa; a large, public-sector (with NGO support), outpatient, HIV treatment facility located within a university-affiliated teaching hospital. General HIV care and ART has been available at Themba Lethu since treatment first became available in public-sector facilities in 2004. Today, Themba Lethu is one of the largest HIV treatment facilities in the country having initiated over 20,000 people living with HIV on ART.[15]

HIV care at Themba Lethu is provided according to national treatment guidelines. Current guidelines, implemented in September 2016, call for treating all those who test HIV positive.[16] However, during the period of study enrollment, people living with HIV were initiated onto ART if their CD4 count fell below 350 cells/mm^3^, were co-infected with tuberculosis, were pregnant or breastfeeding, or if they had a WHO Stage III or IV condition.[17] To monitor the response to ART, viral load testing is conducted at 6 months after ART initiation, 1 year, and then annually thereafter and a CD4 count is conducted at baseline and 12 months post-ART initiation.[17] All information pertinent to patient care, including demographic details, clinical data, prescribed medications, and laboratory results, are captured within an electronic medical record system called TherapyEdge-HIV™ (Advanced Biological Laboratories [ABL] S.A., Luxembourg). Data is entered into TherapyEdge-HIV™ in real-time during the patient encounter and laboratory results are uploaded directly from the National Health Laboratory System.

### Study population

From July to December 2014, we approached HIV-positive adult (≥18 years old) patients at Themba Lethu Clinic who had been on ART between 6 and 18 months, were on a standard first-line ART regimen, had a recent (within the previous 3 months) viral load result available, were able to speak and understand English, and were willing to provide written informed consent for inclusion in the study. The consent process was conducted in English and after reviewing the consent form with the patient, the interviewer asked the patient questions to ensure that the consent form and study procedures were understood (i.e. what will happen if you agree to participate?). If the patient was unsure, the consent form was re-reviewed with the patient until they understood all study procedures.

We selected patients within the 6–18 month time frame in order to be able to assess whether depressive symptoms are associated with virologic response to ART (as a proxy marker of adherence) as, if an association exists, it may be an opportunity for early intervention. During the period of study enrollment, patients with viral loads <400 copies/mL were considered virologically suppressed while those with viral loads >1000 copies/mL were considered at risk of virologic failure. Thus, we included patients from two groups: those whose most recent viral load was >1000 copies/mL (as an indicator of poor adherence) and those whose most recent viral load was <400 copies/mL (as an indicator of good adherence).

### Study procedures

Eligible patients were pre-identified using the TherapyEdge-HIV^TM^ system and recruited by a single patient interviewer on the date of a medical visit (to facilitate referral to a clinician, if necessary). Upon enrollment, the presence of depressive symptoms was evaluated in an interviewer-administered format using the Patient Health Questionnaire (PHQ)-9, a short screening tool that identifies symptoms of depression within the preceding two weeks.[18] The PHQ-9 was recommended for screening for depression in people living with HIV by the South African HIV Clinician’s Society in 2013.[19] Additional information on self-reported prior depression diagnoses and current depression medication, if any, were also collected.

All patients who participated in the study, regardless of the presence or absence of depressive symptoms, were provided with information on depression and how to access mental health services in South Africa, generally, and at the Helen Joseph Hospital, specifically. Patients identified as having any symptoms of depression were referred to their treating clinician for further evaluation and follow-up and there was no further interaction between the patient interviewer and the participant.

### Outcomes

The primary outcome of this study was the presence of depressive symptoms. For each question on the PHQ-9, patients select how many times in the preceding two weeks they have experienced the issue the question addresses: not at all (0 points), several days (1 point), more than half the days (2 points), nearly every day (3 points). The points were then summed across all 9 questions and the standard score of ≥10 was used to indicate depression. The prevalence of depressive symptoms as well as baseline demographic and clinical characteristics are presented as proportions for categorical variables and medians with interquartile ranges (IQR) for continuous variables. Due to the small number of individuals enrolled in this study, and in particular the small number with evidence of depression, we elected to use the median for continuous variables to avoid outliers affecting the means. To quantify the association between baseline demographic and clinical characteristics and the prevalence of depressive symptoms, we present the prevalence difference [PD] with corresponding 95% confidence intervals (CI).

The secondary outcome of interest was to describe outcomes one year after study enrollment. First, to investigate the relationship between depressive symptoms and the result of the next viral load test (as a proxy for adherence), patients were followed from the date of study enrollment until the date of their next viral load result, for a maximum of 12 months post-study enrollment. Second, we also investigated whether retention in care differed between patients with and without depressive symptoms and present the proportion of patients alive and in care one-year after screening.

### Ethical approval

Ethical approval was provided by the Human Research Ethics Committee (Medical) of the University of the Witwatersrand.

## Results

142 individuals were approached to participate in the study; 10 individuals refused to participate and a further 24 did not meet study inclusion criteria, primarily due to not being able to speak and understand English. 108 patients provided informed consent and completed the PHQ-9 with the study interviewer; however, 1 patient could not be identified in the electronic medical record post-enrollment and 10 were found to be ineligible based on their previous viral load results and were excluded from further analysis. At the time of study enrollment, the 97 included patients had been on ART for a median (IQR) of 8.1 (7.3–10.0) months; 61% were female, the median (IQR) age at study enrollment was 38.2 (32.5–42.4) years, and the median (IQR) CD4 count at ART initiation was 154.5 (65–263) cells/mm^3^. The majority (95%) of patients were virologically suppressed with a recent viral load of <400 copies/ml; however, 5 (5%) had recently experienced an elevated viral load (>1000 copies/ml) (Table 1).

**Table 1:** Demographic and clinical characteristics of 97 HIV-positive patients on antiretroviral therapy screened for depressive symptoms at the Themba Lethu Clinic in Johannesburg, South Africa between July and December 2014.

Characteristic	Total		PHQ-9 <10		PHQ-9 ≥10		Prevalence difference (95% CI)	
*Total*	97		90 (92.8%)^1^		7 (7.2%)			
***Sex***								
Male	38		37 (97.4%)		1 (2.6%)		Reference	
Female	59		53 (89.8%)		6 (10.2%)		7.5% (-1.7%-16.8%)	
***Age at initiation***								
Median (IQR)	38.2 (32.5–42.4)		38.3 (32.6–42.4)		36.5 (29.6–44.4)			
<30	14		12 (85.7%)		2 (14.3%)		Reference	
30–39	49		47 (95.9%)		2 (4.1%)		-10.2% (-29.4%-9.0%)	
≥40	34		31 (91.2%)		3 (8.8%)		-5.5% (-26.1%-15.2%)	
***Country of birth***								
South Africa	75		70 (93.3%)		5 (6.7%)		Reference	
Other^2^	22		20 (90.9%)		2 (9.1%)		2.4% (-10.9%-15.7%)	
***Employment status***								
Missing	3		2 (66.7%)		1 (33.3%)		—	
Unemployed	28		26 (92.9%)		2 (7.1%)		1.1% (-10.1%-12.2%)	
Employed	66		62 (93.9%)		4 (6.1%)		Reference	
***Most recent viral load (copies/ml)***								
<400	92		87 (94.6%)		5 (5.4%)		Reference	
>1000	5		3 (60.0%)		2 (40.0%)		34.6% (-8.6%-77.6%)	
***CD4 count (cells/mm***^***3***^***) at ART initiation***								
Median (IQR)	154.5 (65–263)		157 (73–263)		72 (47–83)			
Missing	11		9 (81.8%)		2 (18.2%)		—	
<200	52		48 (92.3%)		4 (7.7%)		4.8% (-4.5%-14.0%)	
≥200	34		33 (97.1%)		1 (2.9%)		Reference	
***BMI (kg/m***^***2***^***) at ART initiation***								
Median (IQR)	21.7 (19.4–26.3)		21.6 (19.4–26.0)		22.1 (18.0–29.0)			
Missing	5		5 (100%)		0 (0.0%)		—	
<18.5	14		12 (85.7%)		2 (14.3%)		10.3% (-8.8%-29.4%)	
18.5–24.9	50		48 (96.0%)		2 (4.0%)		Reference	
≥25	28		25 (89.3%)		3 (10.7%)		6.7% (-6.0%-19.4%)	
***Anaemia (Hb g/dL) at ART initiation***								
Median (IQR) Hb	11.8 (10.3–13.2)		11.9 (10.3–13.2)		10.7 (10.0–13.1)			
Missing	3		3 (100.0%)		0 (0.0%)		—	
None	40		37 (92.5%)		3 (7.5%)		0.1% (-10.7%-10.8%)	
Any^3^	54		50 (92.6%)		4 (7.4%)		Reference	
***First ART regimen***								
TDF-EMT-EFV^4^	82		78 (95.1%)		4 (4.8%)		Reference	
Other^5^	15		12 (80.0%)		3 (20.0%)		15.1% (-5.7%-35.9%)	
***Time on first-line ART (months)***								
Median (IQR)	8.1 (7.3–10.0)		8.2 (7.3–10.0)		7.3 (7.3–15.6)			

^1^Row percentages reported

^2^Other countries: Congo (n = 1), Malawi (n = 1), Mozambique (n = 1), Nigeria (n = 1), Somalia (n = 1), Zambia (n = 1), and Zimbabwe (n = 16)

^3^Any anemia defined as Hemoglobin (Hb) <12 g/dL for women and Hb <13 g/dL for men

^4^Tenofovir (TDF)-Emtricitabine (EMT)-Efavirenz (EFV)

^5^Other ART regimens: TDF-Lamividune (3TC)-EFV (n = 6), TDF-3TC-Nevirapine (NVP) (n = 4), Zidovudine (AZT)-3TC-NVP (n = 1), Stavudine (d4T)-3TC-EFV (n = 4)

### Presence of depressive symptoms

**Fig 1:**
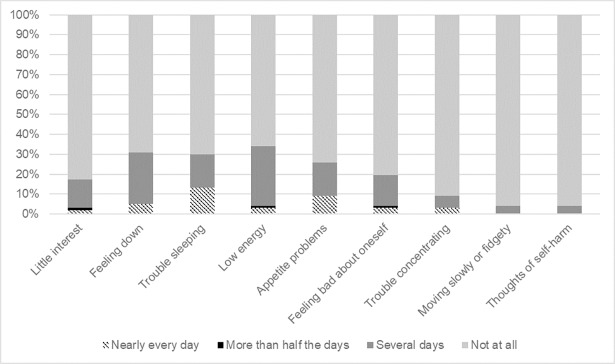
Distribution of responses to the PHQ-9 questionnaire by 97 HIV-infected patients on antiretroviral therapy screened for depressive symptoms at the Themba Lethu Clinic in Johannesburg, South Africa between July and December 2014.

Depressive symptoms greater than minimal (PHQ-9 score ≥10) were identified in 7 (7%) enrolled patients, 4 (4%) of whom were identified as having symptoms of moderate depression (PHQ-9 score: 10–14) and 3 (3%) of whom had symptoms of moderate/severe depression (PHQ: 15–19). None had a score of ≥20 indicating symptoms of severe depression. Regarding the most common symptoms reported, 34% of patients reported problems with low energy in the previous two weeks, 31% reported feeling down, depressed, or hopeless, 30% reported trouble sleeping, and 26% reported appetite problems (Fig 1). Four (4%) patients self-reported being previously diagnosed with depression, one of whom reported current medication use for depression, though none had evidence of current depression on the PHQ-9.

Women (10%) were more likely to report symptoms of depression compared to men (3%; prevalence difference [PD]: 7.5%; 95% CI: -1.7%-16.8%); as were patients under the age of 30 years (14%) compared to those 30–39 years (4%; PD: -10.2%; 95% CI: -29.4%-9.0%) or ≥40 years (9%; PD: -5.5%; -26.1%-15.2%), those with lower CD4 counts at ART initiation (<200 cells/mm^3^ vs ≥200 cells/mm^3^: 8% vs 3%; PD: 4.8%; 95% CI: -4.5%-14.0%), and those with high viral loads (>1000 copies/mL vs. <400 copies/mL: 40% vs. 5%; PD: 34.6%; 95% CI: (-8.6%-77.6%). Patients who were initiated on regimens other than tenofovir (TDF) with emtricitabine (EMT) and efavirenz (EFV) were also more likely to have evidence of depression (20% vs. 5%; PD: 15.1%; 95%: CI: -5.7%-35.9%) but no differences were observed for the presence or absence of anaemia, employment status, or country of birth (Table 1).

### Outcomes one-year after screening

Patients were followed for one-year after screening for depression and 90% were alive and in care at the end of follow-up. Of the 7 patients with a PHQ-9 score ≥10, 6 (86%) were alive and in care one-year after screening and 1 (14%) patient had died. In comparison, 81 (90%) of the 90 patients without evidence of depression were alive and in care one-year after screening, 3 (3%) had died, 4 (4%) were lost to follow-up, and 2 (2%) had transferred to another HIV treatment facility (Table 2).

**Table 2:** One-year treatment outcomes of 97 HIV-positive patients on antiretroviral therapy screened for depressive symptoms at the Themba Lethu Clinic in Johannesburg, South Africa between July and December 2014.

Characteristic	Total	PHQ-9 <10	PHQ-9 ≥10
*Total*	97	90	7
Alive and in care	87 (89.7%)	81 (90.0%)	6 (85.7%)
Died	4 (4.1%)	3 (3.3%)	1 (14.3%)
Lost to follow-up	4 (4.1%)	4 (4.4%)	0 (0%)
Transferred out	2 (2.1%)	2 (2.2%)	0 (0%)

Three (60%) of the 5 patients who had recently experienced an elevated viral load went on to have confirmed virologic failure (2 consecutive viral loads >1000 copies/ml), and switched to a protease-inhibitor based second-line treatment regimen in a median (IQR) of 2.8 (1.2–4.7) months after the second failing viral load. The prevalence of depressive symptoms among those who failed was 67% (2/3) and 0% (0/2) among those patients who went on to suppress.

## Discussion

We observed an overall low prevalence of depressive symptoms (7%) in this population of primarily stable and virologically suppressed HIV-positive patients on ART, which is lower than that of other studies in HIV-positive populations in South Africa (14%-62%),[6–12]. However, we did observe a much higher prevalence of depression (40%) among patients with a recently elevated viral load which may indicate an association between symptoms of depression and poor adherence. We enrolled few patients with a recently elevated viral load, however, and further research is necessary to determine the full extent of this relationship. In addition, we observed similar levels of retention in HIV care one year after screening for depressive symptoms for those with and without evidence of depression. However, few patients with evidence of depression were identified and this finding should be evaluated in a larger patient population.

A recent study conducted among newly-diagnosed, HIV-positive patients in Johannesburg observed a prevalence of depression of 30% using the PHQ-9 and found no association between depressive symptoms and linkage to HIV care or ART initiation.[9] However, patients with depression (as indicated by a PHQ-9 score ≥10), were more likely to perceive their health status as poor (43.6% vs 20.3%) compared to patients without depression and were more likely to have a CD4 count <200 cells/mm^3^ (63.6% vs. 47.8%).[9] Given our observation of a 40% prevalence of depression in patients with a recently elevated viral load, further research to screen for depression at multiple time points along the HIV care continuum (i.e. at HIV diagnosis, ART initiation, and at regular intervals on ART) is needed in order to understand the dynamics between depression, ART use, and HIV treatment outcomes. If an association exists between depression and adherence issues on ART, this work could assist in identifying opportunities for early intervention that could improve HIV treatment outcomes.

Interpretation of our findings should be made in the context of several important factors. Our study population was primarily made up of stable patients on ART for at least 6 months and, thus, our results may not be directly comparable with other research. In addition, this study took place within a well-resourced clinic that focuses solely on HIV care and operates within a university teaching hospital in an urban setting with access to a range of services. Therefore, the experiences of patients in this setting may not be generalizable to patients accessing HIV care in a primary care setting. Furthermore, a number of different tools have been used to evaluate the presence of depressive symptoms in people living with HIV in South Africa with varying levels of sensitivity and specificity and results may not be generalizable across tools.

The results of this study should be viewed in light of several limitations. First, only patients who could speak and understand English were included which may limit generalizability. While English is commonly spoken throughout South Africa, proficiency among Black South Africans (of whom just 2.9% reported English as their first language in the 2011 census) may be limited to those with several years of schooling.[20] Second, our results may be subject to a survivor bias as we only included patients who had been on ART for at least 6 months. If a negative association does exist between depression and retention in care, patients with depression may have left care prior to reaching 6 months on treatment. Third, we had a small sample size with a limited number of patients who had recently experienced an elevated viral load and were unable to fully explore the relationship between depressive symptoms and virologic response. Finally, we utilized an interviewer-administered approach which may be subject to social-desirability bias and a self-administered approach may yield different results.

## Conclusions

This is one of the first studies to assess the prevalence of depression in primarily stable HIV-positive patients on ART in South Africa. We observed a low prevalence of depressive symptoms among virologically suppressed patients while approximately 40% of patients with a recently elevated viral load had evidence of depression. This relationship should be further explored in a larger study and, if confirmed, future work may be warranted to evaluate whether a mental health intervention could treat depression, improve adherence, and prevent the need for switching to more costly second-line treatment regimens in this population.
